# Group treatments for sensitive health care problems: a randomised controlled trial of group versus individual physiotherapy sessions for female urinary incontinence

**DOI:** 10.1186/1472-6874-9-26

**Published:** 2009-09-14

**Authors:** SE Lamb, J Pepper, R Lall, EC Jørstad-Stein, MD Clark, L Hill, J Fereday-Smith

**Affiliations:** 1 Warwick Clinical Trials Unit, Warwick Medical School, University of Warwick, Coventry, CV4 7AL, UK; 2 Health Sciences Research Institute, Warwick Medical School, University of Warwick, CV4 7AL, UK; 3 Physiotherapy Department, George Eliot Hospital, College Street, Nuneaton, Warwickshire, CV10 7DJ, UK; 4 Warwick Hospital, Lakin Road, Warwick, CV34 5BW, UK

## Abstract

**Background:**

The aim was to compare effectiveness of group versus individual sessions of physiotherapy in terms of symptoms, quality of life, and costs, and to investigate the effect of patient preference on uptake and outcome of treatment.

**Methods:**

A pragmatic, multi-centre randomised controlled trial in five British National Health Service physiotherapy departments. 174 women with stress and/or urge incontinence were randomised to receive treatment from a physiotherapist delivered in a group or individual setting over three weekly sessions. Outcome were measured as Symptom Severity Index; Incontinence-related Quality of Life questionnaire; National Health Service costs, and out of pocket expenses.

**Results:**

The majority of women expressed no preference (55%) or preference for individual treatment (36%). Treatment attendance was good, with similar attendance with both service delivery models. Overall, there were no statistically significant differences in symptom severity or quality of life outcomes between the models. Over 85% of women reported a subjective benefit of treatment, with a slightly higher rating in the individual compared with the group setting. When all health care costs were considered, average cost per patient was lower for group sessions (Mean cost difference £52.91 95%, confidence interval (£25.82 - £80.00)).

**Conclusion:**

Indications are that whilst some women may have an initial preference for individual treatment, there are no substantial differences in the symptom, quality of life outcomes or non-attendance. Because of the significant difference in mean cost, group treatment is recommended.

**Trial Registration:**

**Trial Registration numbe**r: ISRCTN 16772662

## Background

Female urinary incontinence (FUI) is a major health problem and has a significant detrimental impact quality of life [1]. Feelings of low self-esteem, embarrassment and helplessness are common[2]. Between 20-50% of women suffer from incontinence at some time in their lives [3,4], and the prevalence of symptoms and demand for services is rising substantially [5]. Pelvic floor exercises delivered in an individual consultation with a physiotherapist are effective in the management of stress-related and urge female urinary incontinence [6-8]. Some physiotherapists advocate the use of group treatments to deliver these treatments, the hypothetical benefits being increased peer-support, mutual self-help, giving and sharing of information, reduction in depression and isolation, increased motivation and compliance with treatment[9]. A large randomised controlled trial (RCT) from the Netherlands showed no difference in the physical responses to treatment between individual and group sessions, but did not consider quality of life, acceptability or cost [10]. If effectiveness can be demonstrated across a range of physical and health related quality of life outcomes, the group approach is potentially more cost-effective, provided that other important components of resource use are not increased, and women are not deterred from attending treatment. The aim of this study was to undertake an appraisal of the acceptability, clinical effectiveness and relative costs of group versus individual treatment sessions in the management of female urinary incontinence. The study used a mixed methods approach, integrating quantitative and qualitative approaches alongside a prospective cost analysis. The qualitative study is reported in an accompanying paper.

## Methods

### Aims

To (i) compare effectiveness in terms of symptoms, quality of life, and costs. (ii) Determine patient preference for group or individual physiotherapy sessions for the management of female urinary incontinence and the association between patient preference and outcome.

### Participants and eligibility

Inclusion criteria were (i) women aged 18 years and over; (ii) able and willing to give informed written consent with an interpreter if necessary; (iii) clinical symptoms of stress and/or urge incontinence. Exclusion criteria were (i) pregnancy; (ii) recent pelvic surgery (less than three months); (iii) history of pelvic malignancy; (iv) current urinary infection; (v) grade III and IV prolapse; (vi) disease of the central nervous system (e.g. multiple sclerosis, cerebrovascular accident) or acute mental illness and dementia; (vi) previous physiotherapy for incontinence within the last 12 months.

Women were recruited from primary and secondary care referrals made to physiotherapy departments of five medium to large sized National Health Service (NHS) trusts in England, UK (detailed at the end of the paper). The study had the approval of Local Research Ethics Committees and written consent was obtained from all participants.

### Outcome measures

Assessments were made prior to treatment (baseline), and then at 6 weeks and 5 months after randomisation. The primary outcomes were the Symptom Severity Index (SSI), with scores ranging from 0 (no severity) to 20 (maximum severity) [11] and the Incontinence-related Quality of Life (IQOL) questionnaire, with scores ranging from 0 (very poor) to 100 (excellent) [12]. The SSI focuses on the physical symptoms of urinary incontinence, and IQOL focuses on the psychological and distress aspects of the condition. Secondary outcomes consisted of health service costs incurred in the follow up period, out of pocket expenses and costs incurred by patients. In addition, patients were asked to rate their perception of response to treatment on a 10-point rating of benefit (from no benefit to maximum benefit). Follow-up data were collected by postal questionnaire.

### Preference

Elicitation of preference was independent of randomisation, and was collected at the time the women were invited to participate in the trial. Women were asked, if they could choose, whether their preference was treatment in a group setting, on an individual basis or no preference.

### Intervention

#### Group treatment

The treatment comprised three one-hour long sessions over a three-week period. Group sizes were anticipated to be approximately 10 women. The first session included an explanation of normal bladder function, causes of stress incontinence, teaching and practice of pelvic floor exercises. The second session included causes of urge incontinence, principles of bladder training, discussion and motivation, practice and progression of pelvic floor exercise including exercises to target fast and slow twitch fibres, with a variation of starting positions. The third session included a bladder quiz to re-enforce knowledge, avoidance of aggravating factors, repetition of pelvic floor exercises, discussion and motivation and safe lifting. Women received an individual assessment prior to the groups, including a pelvic floor examination if indicated. Details of the treatment are available at .

#### Individual treatment

All participants received an assessment using the same protocol as the group session, followed by any of the techniques used in the group sessions, taught on a one to one basis. A maximum of three sessions of one-hour duration was permissible, reflecting the duration of the group sessions. Additional treatments, including electrotherapy, vaginal cones and biofeedback were not included in either treatment protocol.

### Randomisation and blinding

Randomisation was in a ratio of 2:1 (group: individual) to facilitate regular group sessions, and minimise waiting for treatment. Researchers involved with data collection, entry and analysis, were blind to the treatment delivered to each participant. It was not possible to blind the participants or the clinical collaborators delivering the treatment. The allocation sequence was random (computer generated). Allocations were coded and transferred to brown, sealed, sequentially numbered envelopes. The sequential entry of participants was carefully monitored during quality control checks.

### Statistical analysis

#### Sample size

The sample size was estimated a priori, assuming a power of 90% and a significance level of 0.05 to detect a minimally clinically important difference between the groups of 2 points (standard deviation 3.16) on the SSI scale. Allowing for a 20% withdrawal and dropout rate, a minimum sample size of a total of 140 patients was required[13]. No correction was needed for the imbalanced randomisation[13].

#### Statistical and economic analysis

All statistical and economic analyses were intention to treat and statistical significance was claimed at p < 0.05. Baseline characteristics were compared for those who completed the trial and the non-completers. To address potential biases due to incomplete data, two analyses were computed (a) complete case analysis (CCA) and (b) replacing missing observations using multiple imputation[14], combined to give one inference using the rules of Little and Rubin [15]. The imputation dataset was used for sensitivity analysis and produced very similar results to the complete case analysis. The complete case analysis has been reported in this paper. The distributions of the data were checked to ensure parametric assumptions were met. Continuous and categorical data were summarised as mean and standard deviation at each time point. Analysis of covariance was used to assess the treatment effects adjusted for age, baseline value and preference for treatment at baseline. For categorical data, logistic regression was used. A simple economic appraisal was conducted alongside the trial. Cost data were collected by self-completed questionnaire to determine direct costs (i.e. those incurred by the patient, NHS or private healthcare system) and major components of productivity loss (i.e. days of absence from usual occupation). Healthcare costs included in and out-patient hospital appointments and admissions (nurse, consultant or other), visits to the GP, medicines and pads on prescription, personal expenditure on medicines, pads, complementary therapies, and other hygiene products. Unit costs for health care were taken from Department of Health reference costs which were available in 2005 [16]. All costs were standardised to the NHS financial year 2004/2005 which was when the cost-analysis was completed. Costs of days off work were based on average wages in 2004. Owing to the relatively short time frame of the study, discounting was not applied. As the economic analysis was by intention to treat, the cost estimates for group treatment includes the costs associated with providing individual treatments in who were randomised to group treatment but received individual treatment. The costs of non-attendance were included in the analysis using intention to treat principles.

## Results

### Participant flow and characteristics

**Figure 1:**
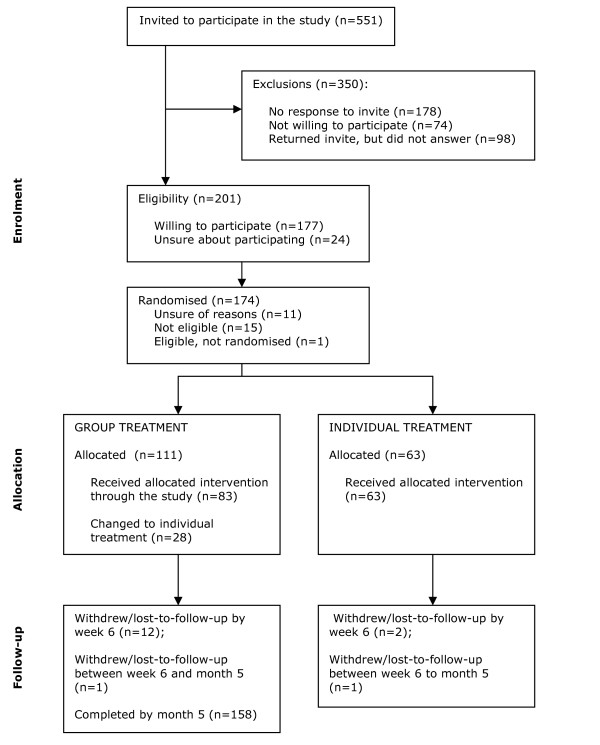
**Consort diagram**.

The flow of participants through the study (consort statement) is given in Figure 1. Between February 2003 and March 2004, 174 eligible women were randomised (111 to group and 63 to individual therapy arms) and 91% (158/174) of women completed the trial. Seven women (4%), all of who were in the group treatment arm, withdrew during the trial - either because of illness (n = 5) or delays in being offered a treatment slot (n = 2). Near equal numbers of women were lost to follow up in each arm (6/111 group treatment; 3/63 in individual treatment). Twenty-eight women in the group therapy sessions received individual as opposed to group sessions. The primary reason for this was the timing of the sessions. Participant characteristics are shown in Table 1, and were well matched in the two arms of the trial. There were no statistically or clinically significant differences between women who completed the trial and those who did not. The number of non-responders between the five referrals centres was borderline significant at 6 weeks (p = 0.041).

**Table 1:** Baseline assessments for each treatment arm

		***Group*** ***(n = 111)***	***Individual (n = 63)***
***Age***	Mean (SD)	49 (10.7)	56 (12.8)

***Height***	Cm (sd)	162.9 (6.61)	161.9 (6.26)

***Weight***	Kg (sd)	75.0 (15.66)	72.2 (14.08)

***First language***	English (%)	109 (98%)	61 (97%)

	Other EU (%)	0 (0%)	1 (2%)

	Asian (%)	2 (2%)	0 (0%)

	Other (%)	0 (0%)	1 (2%)

***Health Status/5***	Mean (SD)	2.7 (1.07)	2.6 (0.8)

***GP appointments in the last year***	Mean (SD)	1.84 (1.59)	1.56 (1.19)

***Education continued after school***	Yes (%)	61 (55%)	35 (56%)

	No (%)	50 (45%)	28 (44%)

***Degree or equivalent qualification***	Yes (%)	36 (32%)	25 (40%)

	No (%)	75 (68%)	38 (60%)

***Employed***	Yes (%)	73 (66%)	33 (52%)

	No (%)	38 (34%)	30 (48%)

***Other limiting illness***	Yes (%)	36 (32%)	21 (33%)

***Years of living with urinary problem***	Mean (SD)	5.8 (6.44)	6.6 (7.71)

***Previous surgical treatment***	Yes (%)	7 (6%)	7 (11%)

	No (%)	103 (93%)	56 (89%)

***SSI (0 - 20)***	Mean (SD)	10.62 (3.96)	10.08 (4.41)

***IQOL (0 - 100)***	Mean (SD)	58.48 (23.01)	61.27 (21.49)

***Previous physiotherapy treatment***	Yes	10 (9%)	7 (11%)

	No	101 (91%)	56 (89%)

***Preference for treatment prior to study ***	Group	12 (11%)	4 (6%)

	Individual	38 (34%)	24 (38%)

	No preference	61 (55%)	35 (55%)

### Treatments received

**Figure 2:**
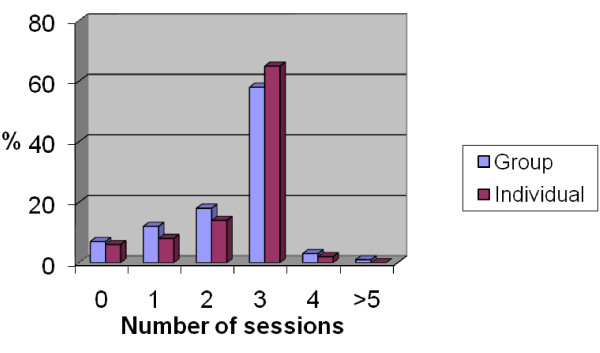
**Percentage of women randomised who attended 0, 1, 2, 3, 4 and > 5 sessions by assignment**.

Five physiotherapists were involved in delivering the intervention, and gave both individual and group treatments. The median number of treatment sessions was 3 in both the individual and group treatment arms. The uptake of treatment was similar across all sites. Adherence with the clinical protocol was good, with only 4% of women receiving more than 3 treatment sessions in the group arm, and 2% in the individual arm (see Figure 2 for treatment attendance). Equal numbers of women did not attend treatment in each arm (6%). The average group size was 7, decreasing to 6.4 and 6.3 women at the second and third groups. Treatments received in either arm were almost identical with over 90% of group and individual sessions containing education, advice and pelvic floor exercises. Bladder re-education was a common component of both individual (78%) and group based treatments (70%). Digital examination was used more frequently in the individual group (9.5% versus 4%).

### Efficacy analysis

#### Disease specific outcomes

The majority of women reported improvements in the SSI and IQOL over the study period, with no statistically or clinically significant differences between the arms of the trial (Additional file 1).

#### Self-rated assessment of treatment benefit

Women in the individual arm reported greater self-rated benefit (data shown in Table 2).

**Table 2:** Number (and percentage) of women who gained benefit from treatment (ranging from 0 to 10) for the follow-up time-points

	**3 months**		**5 months**	
**Benefit gained from treatment (0-10)**	**Group**	**Individual**	**Group**	**Individual**

Missing	15 (13.5%)	8 (12.7%)	12 (10.8%)	3 (4.8%)

0	7 (6.3%)	3 (4.8%)	7 (6.3%)	5 (7.9%)

1	2 (1.8%)	0 (0%)	3 (2.7%)	0 (0%)

2	7 (6.3%)	1 (1.6%)	3 (2.7%)	3 (4.8%)

3	16 (14.4%)	3 (4.8%)	13 (11.7%)	2 (3.2%)

4	9 (8.1%)	5 (7.9%)	7 (6.3%)	3 (4.8%)

5	15 (13.5%)	11 (17.5%)	21 (18.9%)	9 (14.3%)

6	10 (9%)	5 (7.9%)	6 (5.4%)	3 (4.8%)

7	10 (9%)	7 (11.1%)	13 (11.7%)	9 (14.3%)

8	12 (10.8%)	9 (14.3%)	13 (11.7%)	13 (20.6%)

9	2 (1.8%)	1 (1.6%)	6 (5.4%)	6 (9.5%)

10	6 (5.4%)	10 (15.9%)	7 (6.3%)	7 (11.1%)

### Preferences

Just over half of the women (55% in each group) expressed no preference. Of the remainder, the majority expressed a preference for individual treatment and these were near equally allocated to group and individual treatment (Table 1). Preference was associated weakly with more severe symptoms (measured by the SSI). Women who expressed a preference for group treatment tended toward milder symptoms (χ^2 ^= 11.65 p < 0.05). However, using a formal test of interaction, preference was not associated with outcome. Also, preference was not associated with any of the other demographic or baseline characteristics

### Cost analysis

Group treatments cost significantly less (Additional file 2). The average cost to the NHS of the treatments provision was £7.73 per group participant, and £53.37 per participant for individual treatment. When all health care costs were considered (including NHS service costs and patient costs incurred), the average cost per patient was £52.91 (£25.82 to £80.00) lower in the group treatment arm.

## Discussion

It is accepted that education, advice, pelvic floor exercises and bladder re-training are essential and effective in the management of stress and urge urinary incontinence in both younger and older women [17-19]. Other treatments including electrotherapies, biofeedback, vaginal cones offer no substantial advantage over these approaches[8,18] and hence were excluded from both the individual and group treatment arms. By five months symptoms were reduced and incontinence quality of life improved, regardless of the treatment allocation. Responses to treatment in terms of disease specific outcomes were greater in women allocated to the group sessions, although the difference did not reach statistical significance. If left unmanaged, symptoms of incontinence would not resolve spontaneously[20].

The burden of incontinence is well recognised at the population level [5]. Demand for incontinence services is high, and is set to increase substantially due to the combination of population ageing and more women seeking treatment [5]. Significant gains in health related quality of life would occur if effective treatments were both provided to and used by women with incontinence. Group treatments are attractive from the provider perspective, and our study confirms that groups are associated with lower average costs.

Although there was a trend toward greater benefits in terms of health related quality of life in the group sessions, these did not reach statistical significance. This is surprising as lowering psychological burden, which is one of the main components of the IQOL, is one of the postulated mechanisms of group sessions. It is possible that the format of groups was too didactic, and a more interactive approach may have been better.

Being embarrassed about symptoms is a barrier to seeking treatment[21]. Surprisingly, preference for treatments had little association with treatment effectiveness. One explanation is that only a minority of women expressed a preference. Further insight may have been gained by measuring the strength of preference. Women who were randomised against their preference for individual treatment (i.e. to group treatment) were interviewed to ascertain their concerns and to gain insights into improving the acceptability of the service delivery model. These findings are reported in the accompanying manuscript.

Self-rated response to the intervention was somewhat convergent with the disease specific outcomes. This discrepancy is most likely due to the increased and individualised attention that women receiving one to one treatments may have perceived. Attendance at the sessions was similar in both arms of the trial, and further supports the hypothesis that acceptability was equal.

### Strengths and Weaknesses of the Study

Randomised comparisons of different service delivery models are relatively rare. The sample size was calculated a priori, without accounting for any potential clustering or therapist effects. As the loss to follow up rate was smaller than anticipated, the eventual sample size was 158 patients, giving sufficient power to account for the modest clustering effects observed. We cannot imply equivalence of these two approaches; we were not powered to do so, but can confirm that there was no indication of a substantial and systematic difference between the treatments.

The follow up time period was relatively short, although similar to several trials of physiotherapy[22]. We cannot generalise beyond the 5-month follow up period. Future studies should have longer periods of follow up.

We monitored relative costs using self-reported data using a postal questionnaire. Whilst it was not possible to collect detailed costs that can be ascertained during face-to-face interview[23], the use of questionnaires to determine major costs is recognised as a valid technique [24].

## Conclusion

Whilst women or health service providers may have initial reservations about group treatment, there are no substantial differences in the symptom, psychological, or quality of life outcomes. Attendance was very similar between the two arms, allying fears that the groups might intimidate women, and result in non-attendance. Acceptability was explored further to determine how implementation of group treatments might be improved, and is presented in the accompanying paper.

Findings are consistent with our knowledge of the physical effects of physiotherapy for treatment for urinary incontinence[22]. This is the first trial to investigate the claim group treatments may have a psychological effect. The group sessions were no more effective in reducing the burden of psychological symptoms and distress than individual sessions.

Urinary incontinence is a significant public health problem, and group treatments offer one method of offering treatment to large groups of patients. Commissioners and policy makers should feel confident that women will both attend and gain benefit from group sessions.

## Competing interests

The authors declare that they have no competing interests.

## Authors' contributions

The original concept for the study came from JFS and LH. SL designed the trial, secured funding, oversaw the conduct and analysis, and drafted the manuscript. JP was the trial co-ordinator. RL and EJS analysed the data and contributed to drafting the manuscript. MC contributed to the economic analysis. All authors approved the final version of the manuscript.

## Pre-publication history

The pre-publication history for this paper can be accessed here:



## Supplementary Material

Additional file 1 **Complete case analysis**. Summary statistics and covariance analysis of the SSI, IQOL indices and benefit gained from treatment. Click here for file

Additional file 2 **Analysis of resource use and cost data**. Results of economic analysis. Click here for file

## References

[B1] Glew J (1986). Continence. A woman's lot?. Nursing Times.

[B2] Ashworth PD, Hagan MT (1993). The meaning of incontinence: a qualitative study of non-geriatric urinary incontinence sufferers. Journal of Advanced Nursing.

[B3] Hannestad Y, Rortveit G, Sandvik H, Hunskaar S (2000). A community-based epidemiological survey of female urinary incontinence. The Norwegian EPINCONT study. Journal of Clinical Epidemiology.

[B4] Fenner D, Scott JRGR, Karlan BY, Haney AF (2003). Incontinence. Danforth's Obstetrics & Gynecology.

[B5] Luber KM, Boero S, Choe JY (2001). The demographics of pelvic floor disorders: current observations and future projections. American Journal of Obstetrics & Gynecology.

[B6] Berghmans LC, Hendriks HJ, De Bie RA, van Waalwijk van Doorn ES, Bo K, van Kerrebroeck PE (2000). Conservative treatment of urge urinary incontinence in women: a systematic review of randomized clinical trials. BJU International.

[B7] Fantl JA, Wyman JF, McClish DK, Harkins SW, Elswick RK, Taylor JR, Hadley EC (1991). Efficacy of bladder training in older women with urinary incontinence. JAMA.

[B8] Wallace SA, Roe B, Williams K, Palmer M (2004). Bladder training for urinary incontinence in adults. [see comment] [update of Cochrane Database Syst Rev. 2000;(2):CD001308 PMID: 10796768]. Cochrane Database of Systematic Reviews.

[B9] Cook T (2001). Group treatment of female urinary incontinence: literature review. Physiotherapy.

[B10] Janssen CC, Lagro-Janssen AL, Felling AJ (2001). The effects of physiotherapy for female urinary incontinence: individual compared with group treatment. BJU International.

[B11] Black N, Griffiths J, Pope C (1996). Development of a symptom severity index and a symptom impact index for stress incontinence in women. Neurourol Urodyn.

[B12] Uebersax JS, Wyman JF, Shumaker SA, McClish DK, Fantl JA (1995). Short forms to assess life quality and symptom distress for urinary incontinence in women: the Incontinence Impact Questionnaire and the Urogenital Distress Inventory. Continence Program for Women Research Group. Neurourology & Urodynamics.

[B13] Machin D, Campbell M, Fayers P, Pinol A (1997). Sample size tables for clinical studies Oxford edition.

[B14] Schafer J (1997). Analysis of Incomplete Multivariata Data.

[B15] Little R, Rubin D (1987). Statistical Analysis with Missing data.

[B16] Netten A, Curtin L (2005). Unit cost of Health and Social Care.

[B17] Burgio KL, Goode PS, Locher JL, Umlauf MG, Roth DL, Richter HE, Varner RE, Lloyd LK (2002). Behavioral training with and without biofeedback in the treatment of urge incontinence in older women: a randomized controlled trial. [see comment]. JAMA.

[B18] Goode PS, Burgio KL, Locher JL, Roth DL, Umlauf MG, Richter HE, Varner RE, Lloyd LK (2003). Effect of behavioral training with or without pelvic floor electrical stimulation on stress incontinence in women: a randomized controlled trial. [see comment]. JAMA.

[B19] Holroyd-Leduc JM, Straus SE (2004). Management of urinary incontinence in women: clinical applications. JAMA.

[B20] Bo K, Talseth T, Holme I (1999). Single blind, randomised controlled trial of pelvic floor exercises, electrical stimulation, vaginal cones, and no treatment in management of genuine stress incontinence in women. BMJ.

[B21] Roe B, Doll H, Wilson K (1999). Help seeking behaviour and health and social services utilisation by people suffering from urinary incontinence. International Journal of Nursing Studies.

[B22] Hay-Smith EJC, Dumoulin C (2006). Pelvic floor muscle training versus no treatment, or inactive control treatments, for urinary incontinence in women. Cochrane Database of Systematic Reviews.

[B23] Dowell CJ, Bryant CM, Moore KH, Simons AM (1999). Calculating the direct costs of urinary incontinence: a new test instrument. BJU International.

[B24] Brink M van den, Hout WB van den, Stiggelbout AM, Putter H, Velde CJH van de, Kievit J (2005). Self-reports of health-care utilization: diary or questionnaire?. International Journal of Technology Assessment in Health Care.

