# Supplementary material for: Group treatments for sensitive health care problems: a randomised controlled trial of group versus individual physiotherapy sessions for female urinary incontinence
Source: BMC Womens Health. 2009 Sep 14;9:26. doi: 10.1186/1472-6874-9-26 (PMC2754423; doi:10.1186/1472-6874-9-26)
Supplement: Additional file 1 — Complete case analysis. Summary statistics and covariance analysis of the SSI, IQOL indices and benefit gained from treatment. [file 1472-6874-9-26-S1.doc]

Additional file 1 - Complete case analysis: Summary statistics and covariance analysis of the SSI, IQOL indices and benefit gained from treatment

|  | *Unadjusted summary statistics* | | | | *Covariance analysis*2* | |  |
| --- | --- | --- | --- | --- | --- | --- | --- |
|  | ***Group*** | | ***Individual*** | | ***Group*** | ***Individual*** |  |
| ***Instrument*** | ***N*** | ***Mean (sd)*** | ***N*** | ***Mean (sd)*** | ***Adjusted Mean (se)*** | | ***P**** |
| **Symptom Severity Index** |  |  |  |  |  |  |  |
| 6 weeks | 81 | 9.43 (4.40) | 49 | 9.39 (4.77) | 9.02 (0.41) | 9.47 (0.51) | 0.4444 |
| 5 months | 76 | 8.43 (4.19) | 49 | 8.43 (5.16) | 8.38 (0.50) | 8.64 (0.63) | 0.7179 |
| Baseline to 6 week | 79 | 1.53 (3.33) | 48 | 0.60 (3.12) | 1.25 (0.34) | 1.38 (0.49) | 0.8318 |
| Baseline to 5 months | 74 | 2.35 (3.35) | 47 | 1.77 (4.40) | 2.34 (0.44) | 1.71 (0.57) | 0.3865 |
| **IQOL** |  |  |  |  |  |  |  |
| 6 weeks | 99 | 66.89 (23.27) | 57 | 66.67 (21.29) | 67.77 (1.66) | 65.27 (2.10) | 0.3571 |
| 5 months | 85 | 73.08 (19.55) | 52 | 73.65 (21.52) | 73.26 (2.10) | 71.79 (2.73) | 0.6414 |
| Baseline to 6 week | 90 | -9.72 (16.35) | 57 | -4.99 (17.61) | -9.11 (1.77) | -7.47 (2.30) | 0.5794 |
| Baseline to 5 months | 85 | -15.94 (16.76) | 52 | -11.74 (22.80) | -14.40 (1.75) | -14.81 (2.50) | 0.8941 |
| **Benefit gained from treatment (0-10)** |  |  |  |  |  |  |  |
| 6 weeks | 96 | 4.99 (2.68) | 55 | 6.29 (2.69) | 4.95 (0.28) | 6.40 (0.38) | 0.0029 |
| 5 months | 99 | 5.41 (2.41) | 60 | 6.30 (2.88) | 5.38 (0.29) | 6.34 (0.38) | 0.0462 |
| **How would you rate your urinary problems or incontinence now, compared to before treatment** |  |  |  |  |  |  |  |
| 6 weeks | 84 | 2.29 (0.70) | 53 | 2.23 (0.78) | 2.29 (0.08) | 2.26 (0.11) | 0.7929 |
| 5 months | 85 | 2.27 (0.86) | 52 | 2.13 (1.01) | 2.28 (0.10) | 2.13 (0.13) | 0.3816 |

*^2^- Difference between treatments adjusted for baseline SSI/IQOL, age and preference of treatment

* - p value denotes significance of the comparison between group and individual treatments, using the Wald’s test statistic from the linear regression models (adjusting for covariates)

Note: The SSI ranges from 0 (no severity) to 20 (maximum severity). The Incontinence-related Quality of Life ranges from 0 (very poor) to 100 (excellent).
