# Supplementary material for: Group treatments for sensitive health care problems: a randomised controlled trial of group versus individual physiotherapy sessions for female urinary incontinence
Source: BMC Womens Health. 2009 Sep 14;9:26. doi: 10.1186/1472-6874-9-26 (PMC2754423; doi:10.1186/1472-6874-9-26)
Supplement: Additional file 2 — Analysis of resource use and cost data. Results of economic analysis. [file 1472-6874-9-26-S2.doc]

Additional file 2 - Analysis of resource use and cost data

|  | *Group*  *Mean (SD) n=111* | *Individual*  *Mean (SD) n= 63* | *Mean difference* |
| --- | --- | --- | --- |
| Intervention costs | £7.73 per participant | £53.37 per participant | -£45.64 (-£40.81 to -£50.47) |
| Resource use during follow up Number of days hospitalisation  Number of prescriptions for medicines or pads  Number of medicines or pads bought over the counter  Mean number of pads purchased per person  **National Health Service costs**  Hospitalisation  Prescription costs for bladder symptoms Costs incurred Over the counter medications  Incontinence pads  Opportunity costs (employment)  Costs of medicines | 1 (0.9%)  12  93  131  £3.40 per participant  £3.36 per participant  £19.77 per participant  £13.83 per participant  £2.57 per participant  £0.11 per participant | 2 (3.2%)  8  57  98    £11.98 per participant  £2.79 per participant  £23.26 per participant  £12.68 per participant  £3.01 per participant  £0.96 per participants | -£8.58 (-£32.99 to £15.83)  -£0.57 (-£3.04 to £4.18)  -£3.49 (-£2.65 to £4.33)  £1.15 (-£7.04 to £9.34)  -£0.44 (-£7.43 to £6.55)  -£0.85 (-£1.01 to -£0.69) |
| Average overall cost | £50.77 per participant | £108.05 per participant | -£57.28 (-£84.37, -30.19) |
